# Development of online-storage inner-recycling counter-current chromatography for the preparative separation of complex components of alkylphenols from sarcotesta of *Ginkgo biloba* L.

**DOI:** 10.1039/c8ra05618h

**Published:** 2018-10-05

**Authors:** Daijie Wang, Xiangyun Song, Huijiao Yan, Mengmeng Guo, Ruiming Fu, Hailong Jiang, Heng Zhu, Xiao Wang

**Affiliations:** Key Laboratory of TCM Quality Control, Shandong Analysis and Test Center, Qilu University of Technology (Shandong Academy of Sciences) Jinan Shandong 250014 People's Republic of China wxjn1998@126.com +86-0531-8260-5304; College of Food Science and Engineering, Shandong Agricultural University Taian Shandong 271018 People's Republic of China

## Abstract

High-speed counter-current chromatography (HSCCC) is becoming an effective and non-absorptive separation method from natural products. Due to the insufficient separation efficiency, it is challenging to separate complex components, especially for compounds with similar *K*_D_ values. In this study, a novel and effective online-storage inner-recycling CCC method was used to separate alkylphenols from the sarcotesta of *Ginkgo biloba* L. A two-phase solvent system of *n*-heptane/ethyl acetate/methanol/acetic acid (5 : 4 : 1 : 1, v/v) was used for HSCCC separation of 500 mg crude extracts. After the inner-recycling of two fractions coupled with pre-HPLC, five main ginkgolic acids (C13:0, C15:1, C17:2, C15:1, C17:1) coupled with bilobol (C15:1) and a mixture were obtained from a non-stop separation using a storage loop and two six-way valves. This novel method was also evaluated and predicted by formula derivation. This method could be an effective, rapid, and simple approach to separate alkylphenols from the sarcotesta of *G. biloba*.

## Abstract 2

High-speed counter-current chromatography (HSCCC) is becoming an effective and non-absorptive separation method from natural products.

## Graphical abstract

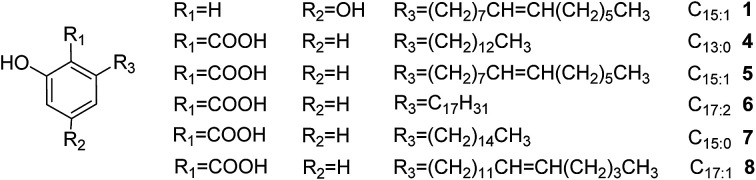

## 1 Introduction

*Ginkgo biloba* L., one of the most famous medicinal plants in the world, is considered as a living fossil and has existed on Earth for over 200 million years.^1^ As a famous medicine and dietary supplement, extracts obtained from the leaves and seeds of *G. biloba* have played an important role in the treatment of Alzheimer's dementia, protecting the hippocampal neurons and improving cognitive performance and social function.^2,3^ In previous studies, the main medicinal components of *Ginkgo biloba* have been identified as terpene trilactones (ginkgolides A, B, and C and bilobalide) and flavonoids (quercetin, kaempferol, and isorhamnetin).^4^ Its alkylphenols, mainly ginkgolic acids (GAs), on the other hand, are considered to be toxic, mutagenic, and allergenic at levels of less than 10 μg g^−1^.^5^ Recently, pharmacological research on alkylphenols has attracted more attention. Alkylphenols show beneficial effects, including anti-cancer, anti-parasitism, anti-bacterial, and molluscicidal activity,^6–9^ and most especially enzyme-inhibiting functions, such as of HIV protease, fatty acid synthase, tyrosinase, glycerol-3-phosphatase, and dehydrogenase, as well as protein SUMOylation.^10–12^ Alkylphenols are abundant in *G. biloba*, especially in the sarcotesta of ginkgo seeds (over 4% GAs, w/w).^13^ Due to the broad pharmacological effects, abundant resources, and quality control of *G. biloba*, it is necessary to establish an efficient method for the isolation and purification of these alkylphenols.

**Fig. 1:**
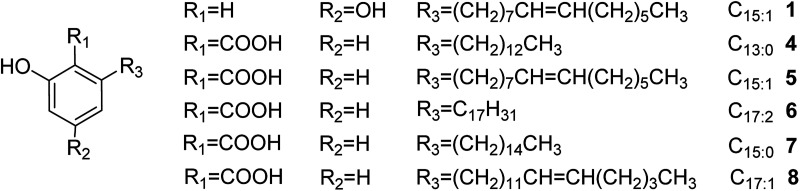
Chemical structures of the separated alkylphenols.

Alkylphenols are recovered as mixtures of a series of homologues. They bear C_13_–C_17_ hydrophobic chains at the 6-position and 0–3 side-chain double bonds (Fig. 1).^14^ Because of the long chains, alkylphenols are low polarity with high hydrophobicity. There have been several reports on the separation of alkylphenols by silica gel or silicone oil chromatography, C_18_–Ag(i)-loaded cation-exchange chromatography, and reversed-phase C_18_ and C_18_ HPLC.^15–17^ The traditional silica gel separation method is tedious and time-consuming and requires large amounts of solvents. The separation is thus expensive, and rigorous sample preparation is required. Moreover, molecules with long hydrophobic chains are liable to be strongly adsorbed on reversed-phase columns. Therefore, an efficient isolation and purification method is urgently needed for improved separation of alkylphenols.

High-speed counter-current chromatography (HSCCC) is a continuous liquid–liquid partition technique that does not require a solid support. As a novel chromatographic technique, it has many advantages, such as the elimination of irreversible adsorption, high sample recovery, low risk of sample denaturation, and large preparative capacity.^18,19^ Recently, it has gradually become a useful tool for the preparative isolation and purification of various natural products.^20,21^

There are also some disadvantages in HSCCC, such as insufficient understanding of its principles, complex selection of solvent system, difficulty in gradient elution and insufficient number of theoretical plates. Especially due to the insufficient number of theoretical plates compared with HPLC, it is difficult to separate similar compounds, especially those with similar *K*_D_ values, by 1D HSCCC. The traditional approach for separating compounds with similar *K*_D_ values has been to increase the separation time, resulting in increased peak broadening and unacceptable consumption of organic solvents. Recently, 2D/multi-D HSCCC methods have been developed to increase peak resolution. The various modes available to date include tandem HSCCC,^22^ off-line 2D/multi-D CCC,^23^ on-line inner-recycling CCC,^24^ and online-storage recycling CCC.^25^ Inner-recycling CCC provides an excellent solution for the separation of compounds with similar *K*_D_ values. It can greatly reduce the amount of solvent used. Coupled with the online-storage mode, it is suitable for the separation of complex components in natural products with similar *K*_D_ values. Herein, an online-storage recycling CCC method is introduced for the separation of alkylphenols with long hydrophobic chains obtained from the sarcotesta of *G. biloba.* To the best of our knowledge, this is the first report on the use of the online-storage and inner-recycling CCC technique for separating such alkylphenols (Fig. 1).

## 2 Materials and methods

### 2.1 Reagents and materials

*n*-Hexane, *n*-heptane, ethyl acetate, methanol, acetic acid and petroleum ether (60–90 °C) used for the preparation of crude extract and CCC separations were analytical grade (Sinopharm Chemical Reagent Co., Ltd, Shanghai, China). HPLC-grade methanol and acetic acid were purchased from the Fisher Company (Fairlawn, NJ, USA). The water used was deionized by an osmosis Milli-Q system (Millipore, Bedford, MA, USA). Reverse osmosis Milli-Q water (Millipore, USA) was used.

Fresh sarcotesta of *G. biloba* was obtained from ginkgo trees in the city of Jinan (Shandong, China) and identified by Dr Jia Li (College of Pharmacy, Shandong University of Traditional Chinese Medicine). A voucher specimen (2016100701) has been deposited at Shandong Analysis and Test Center.

### 2.2 Apparatus

The HSCCC equipment was a TBE-300C (Shanghai, Tauto Biotech, China) with three multilayer coil separation columns of 300 mL (the diameter of the PTFE tube was 2.6 mm) as well as a 20 mL manual sample loop. The HSCCC apparatus was equipped with four other instrument modules, including a TBP-5002 constant-flow pump (Tauto Biotechnique, Shanghai, China), a 8823A-UV Monitor at 254 nm (Beijing Emilion Technology, Beijing, China), a Model 3057 portable recorder (Yokogawa, Sichuan Instrument Factory, Sichuan, China), and a DC-0506 low constant temperature bath (Tauto Biotechnique, Shanghai, China) to maintain the temperature at 25 °C. HPLC separation was performed on a Waters 600 system consisting of a Waters 600 pump, a Photodiode Array Detection (PDA) detector, and an automatic sample injection with Waters Symmetry C_18_ column (250 mm × 4.6 mm, i.d. 5 μm, USA). The storage loop was a Teflon pipeline with an inner diameter and outer diameter of 2 mm and 3 mm, respectively.

### 2.3 Preparation of crude extract

The fresh sarcotestas were firstly separated from seeds and dried in the shade at room temperature. Then 1.5 kg of dry sarcotesta was extracted three times with petroleum ether (10 L). The combined extracts were concentrated under reduced pressure at 40 °C to obtain the crude extract (74 g).

### 2.4 Selection of solvent system

In the present study, a series of two-phase solvent systems in various ratios were tested for their partition capabilities. The partition coefficients (*K*_D_-values) of the target compounds were determined by HPLC as follows. Five milliliters of each phase of the equilibrated two-phase solvent system was added to approximately 10 mg of the crude extract. The test tube was shaken vigorously for 1 min. After the upper and lower phase had separated fully, 1 mL of each layer was removed and dried with nitrogen. The residue was dissolved in 1 mL methanol and analyzed by HPLC. The *K*_D_-values of the target compounds were calculated according to the equation *K* = *A*_U_/*A*_L_, where *A*_U_ was the peak area of the target compound in the upper phase, while *A*_L_ was that of the lower phase.

### 2.5 Preparation of solvent systems and sample solutions

For HSCCC separation, a two-phase solvent system consisting of *n*-heptane/ethyl acetate/methanol/acetic acid (5 : 4 : 1 : 1, v/v) was placed in a separating funnel. After shaking vigorously, the solution was left to stand for a few minutes and divided into two phases for the experiment. The upper layer served as the stationary phase, while the lower layer served as the mobile phase. A sample of crude extract (500 mg) was dissolved in 20 mL of a mixture of the upper and lower phases (1 : 1, v/v).

### 2.6 Separation procedure

#### 2.6.1 Online-storage inner-recycling CCC structure

**Fig. 2:**
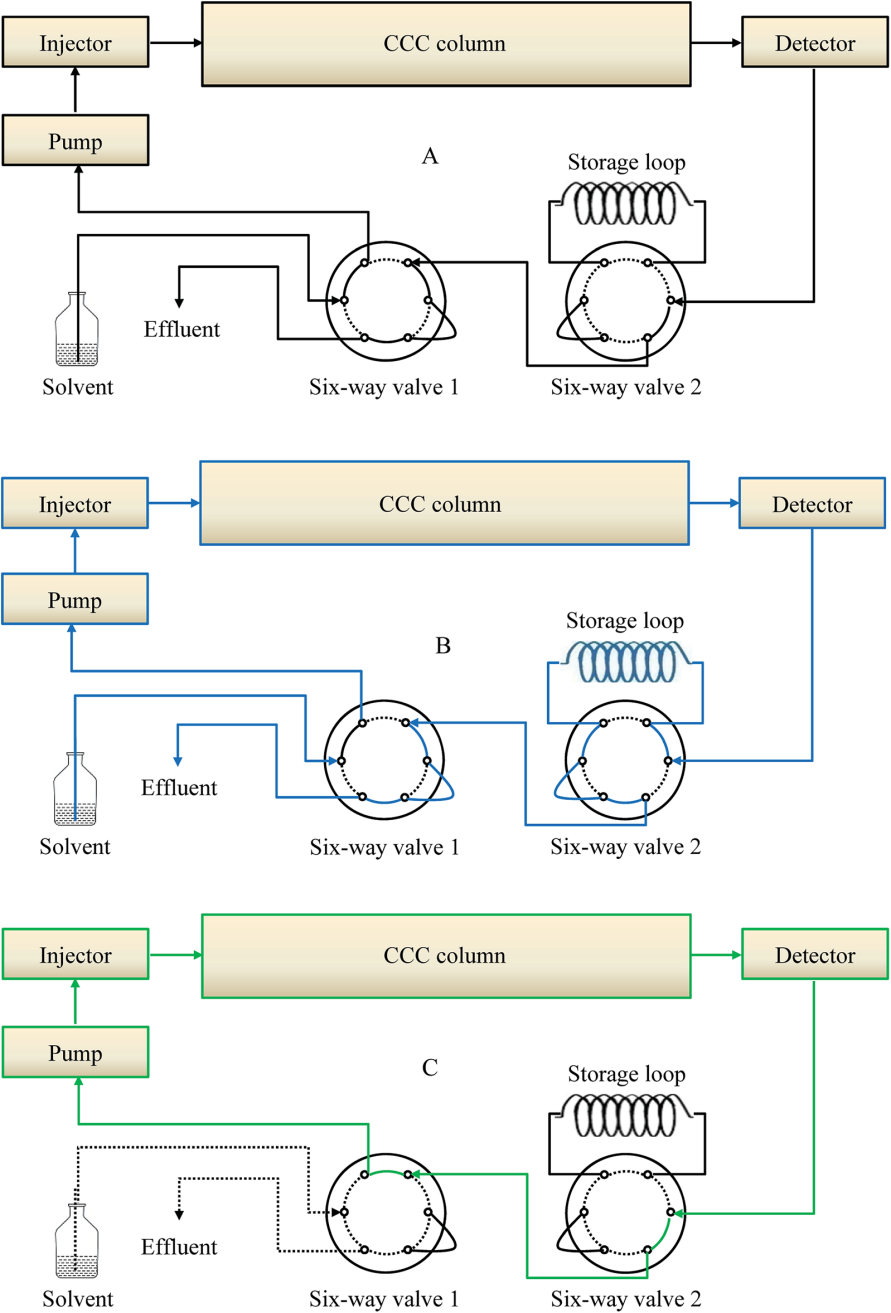
Schematic diagrams of the online-storage inner-recycling CCC separation. (A) Schematic diagrams; (B) online-storage mode; (C) inner-recycling separation mode.

The online-storage inner-recycling CCC set-up is shown in Fig. 2. Two additional six-way valves and a storage loop were added compared with conventional CCC. Six-way valve 1 was introduced between the pump and the solvent bottle to enable switching between collection and inner-recycling modes. Six-way valve 2 was introduced between the detector and the first six-way valve to enable switching to online storage. There were three stages, namely collection, online-storage, and inner-recycling. In the first stage, the six-way valve was in the position for collection (Fig. 2A). This stage encompassed establishing the hydrodynamic equilibrium, sample solution loading, and sample eluent collection. In the second stage of online storage (Fig. 2B), the target sample eluent was fully collected in the storage loop. The third stage involved the inner-recycling mode (Fig. 2C).

#### 2.6.2 Conventional CCC separation

For conventional CCC separation, the six-way valve was switched to enable the collection mode (Fig. 2A). The column of the CCC instrument was first completely filled with the upper phase at 20 mL min^−1^ in head-to-tail elution mode. The sample solution of the GAs extract was then injected with the manual sample loop. The lower phase was pumped into the head of the CCC column at 2.0 mL min^−1^, during which the apparatus was rotated at 800 rpm in a clockwise manner. The separation temperature was set at 25 °C. The effluents were continuously monitored at 254 nm by means of a portable recorder. Four fractions (Fr.) were obtained in the one-step separation. In Fr. III, the three compounds 4, 5, and 6 with similar *K*_D_ values were co-eluted, and compounds 7 and 8 were co-eluted in Fr. IV.

#### 2.6.3 Online-storage recycling CCC separation

Until the time when Fr. III was introduced into the storage loop, the process was the same as that of conventional CCC separation (Fig. 4A and B). When the sample eluent of Fr. III was at the tail end of CCC, the separation mode was switched to the online-storage mode (Fig. 4C). After all of the target fractions had been collected in the storage loop, the six-way valve was turned so as to enable the inner-recycling mode (Fig. 4D). In this stage, target compounds 7 and 8 were completely separated through six inner-recycling procedures. Six-way valve 1 was then switched to enable the collection mode. Compounds 4 and 5 were collected in test tubes. The final step was to separate Fr. III. Six-way valves 1 and 2 were switched and Fr. III was guided to be subjected to the inner-recycling mode (Fig. 4E). In this stage, the target compounds were separated through nine inner-recycling procedures and collected in test tubes. The retention of the stationary phase was defined as the stationary phase relative to the total column capacity after separation.

**Fig. 3:**
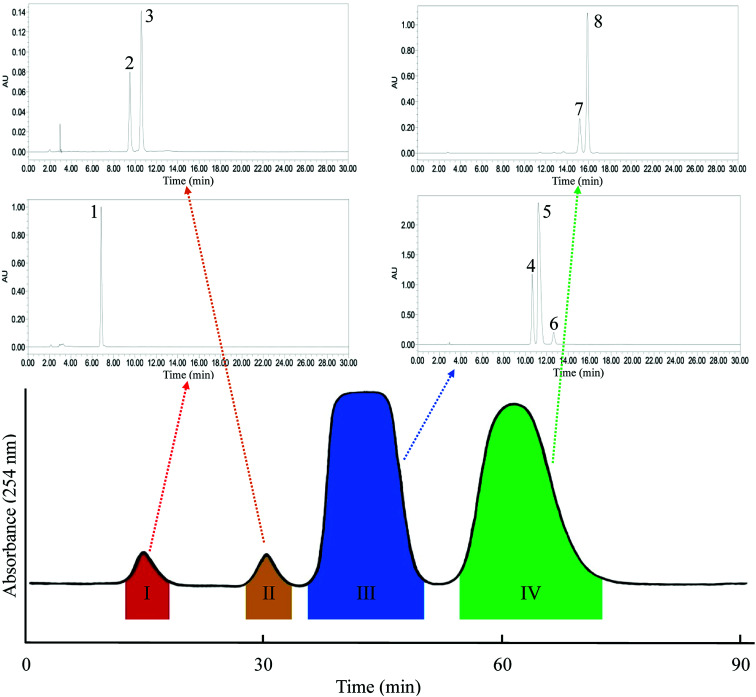
HSCCC chromatogram of one-step separation mode. Solvent system: *n*-heptane/ethyl acetate/methanol/acetic acid (5 : 4 : 1 : 1, v/v); flow-rate: 2.0 mL min^−1^, detection wavelength: 254 nm.

**Fig. 4:**
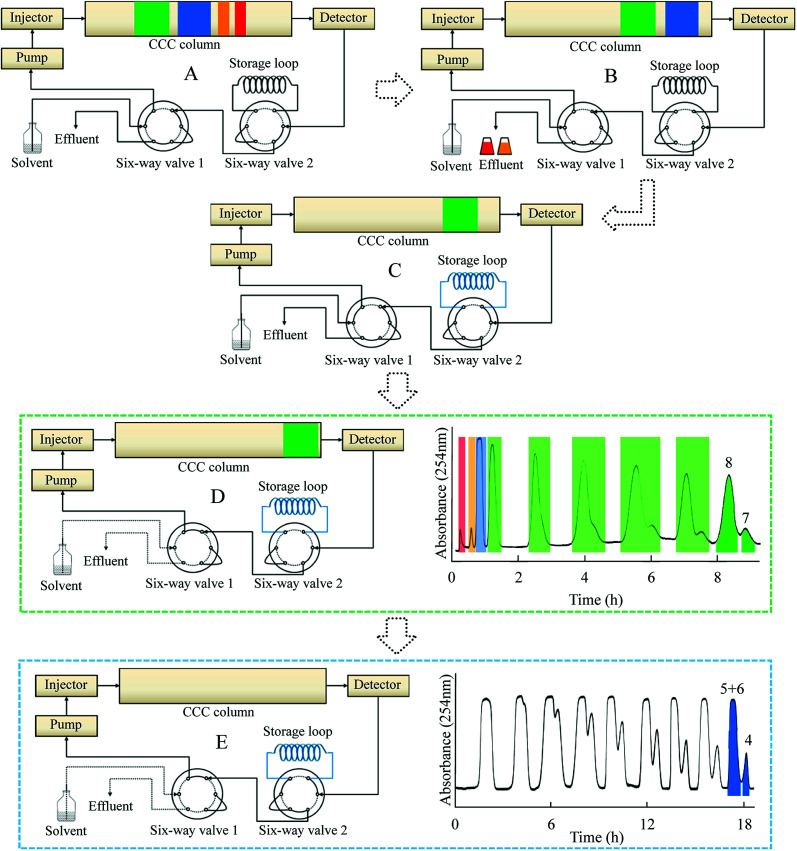
Schematic diagram of online-storage inner-recycling CCC separation procedure. Solvent system: *n*-heptane/ethyl acetate/methanol/acetic acid (5 : 4 : 1 : 1, v/v); flow-rate: 2.0 mL min^−1^, detection wavelength: 254 nm. (A) Separation procedure, (B) separation of Fr. I and II in Fig. 3, (C) online-storage of Fr. III, (D) inner-recycling separation of Fr. IV, (E) inner-recycling separation of Fr. III.

### 2.7 HPLC analysis

HPLC analyses of the extract and CCC fractions were performed on Waters 600 HPLC equipment with a C_18_ column (Waters Symmetry, 5 μm, 4.6 mm × 250 mm, i.d.). The mobile phase was methanol and 0.5% aqueous solution of acetic acid (92 : 8, v/v) with a flow-rate of 1.0 mL min^−1^ and a wavelength of 310 nm.

### 2.8 Structural identification

The separated compounds were identified by ESI-MS and ^1^H and ^13^C NMR spectrometries. ESI-MS analyses were performed on an Agilent 6520 Q-TOF instrument (Agilent, Santa Clara, CA, USA). NMR spectra were performed on a Bruker AV-400 spectrometer (Bruker BioSpin, Rheinstetten, Germany) with CDCl_3_ as solvent, and chemical shifts (*δ*) are expressed in parts per million (ppm) coupled with constant (*J*) in Hz.

## 3 Results and discussion

### 3.1 Selection of the HSCCC solvent systems

For HSCCC separation, a suitable two-phase solvent system was crucial. A suitable solvent system requires an appropriate partition coefficient as well as good sample solubility.^26^ Generally, the most suitable range of *K*_D_ values in HSCCC separation is between 0.5 and 2. A higher *K*_D_ value may lead to excessively broad peaks and extended elution times, whereas a lower *K*_D_ value might lead to poor peak resolution. Additionally, the separation factor (*α*) (*α* = *K*_2_/*K*_1_, *K*_2_ > *K*_1_) is an important parameter. If the *α* value is greater than 1.5, adjacent chromatographic peaks can be completely separated.^27^ A series of mixed-solvent systems was tested, including ethyl acetate/*n*-butanol/water (4 : 1 : 5, v/v), *n*-hexane/ethyl acetate/methanol/water (5 : 2 : 5 : 2, v/v), chloroform/methanol/water (4 : 3 : 2, v/v), and *n*-heptane/ethyl acetate/methanol/water (5 : 4 : 1 : 1, v/v). When ethyl acetate/*n*-butanol/water (4 : 1 : 5, v/v) and *n*-hexane/ethyl acetate/methanol/water (5 : 2 : 5 : 2, v/v) were used, the GAs were mainly distributed in the upper phase, giving *K*_D_ values far greater than 1, making them difficult to elute. When chloroform/methanol/water (4 : 3 : 2, v/v) was used, the GAs were mainly distributed in the chloroform of the lower phase, such that they would be rapidly eluted with no peak resolution. When *n*-heptane/ethyl acetate/methanol/acetic acid (5 : 4 : 1 : 1, v/v) was used, appropriate *K*_D_ values in the range 0.89 to 1.53 were obtained (Table 1). However, as also shown in Table 1, the *K*_D_ values of GA 13:0, GA 15:1, and GA 17:2 were 0.96, 0.89, and 0.90, respectively. The separation factors *K*_3_/*K*_2_ and *K*_1_/*K*_2_ were 1.01 and 1.08. The *K*_D_ values of GA 15:0 and GA 17:1 were 1.53 and 1.41, and the separation factor *K*_4_/*K*_5_ was 1.09. These close *K*_D_ values indicate that the relevant compounds would be difficult to separate in conventional one-step separation mode. Thus, online-storage recycling CCC mode was used for further HSCCC separation.

**Table 1:** The *K*_D_-values of GAs in HSCCC separation with different solvent systems

Solvent system	*K*_D_ *-*values of GAs				
	13:0	15:1	17:2	15:0	17:1
EtOAc/*n*-BuOH/H_2_O (4 : 1 : 5, v/v)	267.3	238.0	181.7	205.2	301.1
*n*-hexane/EtOAc/MeOH/H_2_O (5 : 2 : 5 : 2, v/v)	25.3	29.6	43.1	61.6	69.6
CHCl_3_/MeOH/H_2_O (4 : 3 : 2, v/v)	<0.01	<0.01	<0.01	<0.01	<0.01
Heptane/EtOAc/MeOH/HOAc (5 : 4 : 1 : 1, v/v)	0.96	0.89	0.90	1.53	1.41

### 3.2 Separation of the alkylphenols

According to basic chromatographic theory, a longer CCC column would result in a higher number of theoretical plates. This may be expressed by the following equation:

1 (*R*_1_/*R*_2_)^2^ = *N*_1_/*N*_2_ = *L*_1_/*L*_2_

where *R* is the resolution, *N* is the total number of theoretical plates, and *L* is the length of the column.^28^

For a particular CCC instrument, the number of theoretical plates in a one-step separation is limited. Compared with HPLC, the number of theoretical plates of HSCCC is low, resulting in inferior separation. Inner-recycling CCC is an improvement on conventional HSCCC, whereby a six-way valve is designed to form a closed loop. Coupled with one or more storage loops, it becomes very convenient to separate complex constituents, especially compounds with similar structures and *K*_D_ values in natural products. Compared with the conventional one-step separation mode, it has many advantages, such as much lower organic solvent consumption and enhanced numbers of theoretical plates and peak resolution, while retaining a simple set-up and ease of operation.

Fig. 3 shows a one-step HSCCC separation with the solvent system *n*-heptane/ethyl acetate/methanol/acetic acid (5 : 4 : 1 : 1, v/v), and the result is consistent with the *K*_D_ values listed in Table 1. The main GAs in *G. biloba* were enriched in two fractions. Fr. III contained three GAs, namely C13:0 (peak 4 in Fig. 3), C15:1 (peak 5 in Fig. 3), and C17:2 (peak 6 in Fig. 3). Fr. IV contained C15:1 (peak 7 in Fig. 3) and C17:1 (peak 8 in Fig. 3). The resolution (*R*_s_) of Fr. III and IV was 1.40 as calculated with eqn (3). However, the *R*_s_ of the main compounds within the two fractions was much less than 1 for the subsequent inner-recycling separation. Additionally, two minor components of Fr. I and Fr. II were obtained. Fr. I (10.2 mg) was finally identified as bilobol (C15:1) with purity >98%, as determined by HPLC. Fr. II was identified as a mixture of two compounds, with molecular weights of *m*/*z* 344 and 370, as determined by HPLC-TOF-MS.

**Fig. 5:**
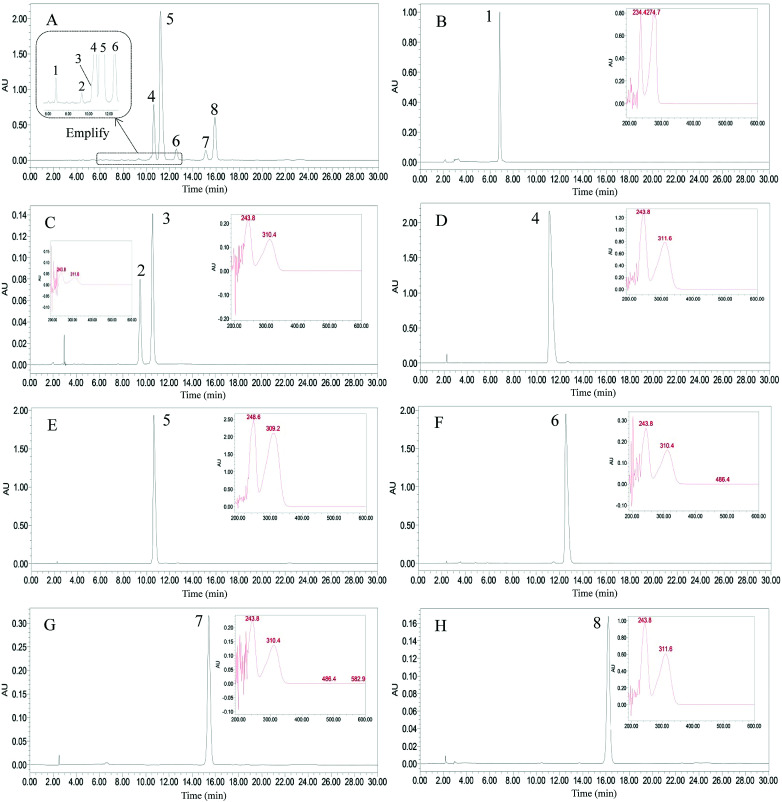
HPLC chromatograms of the crude extract and the isolated alkylphenols. (A) Crude extract, (B) bilobol (15 : 1), (C) Fr. II in Fig. 3, (D) ginkgoneolic acid (GA 13:0), (E) ginkgolic acid (GA 15:1), (F) ginkgolic acid C17:2 (GA 17:2), (G) hydroginkgolic acid (GA 15:0), (H) ginkgolic acid C17:1 (GA 17:1). Experimental conditions: waters symmetry C_18_ column (5 μm, 4.6 mm × 250 mm, i.d.,); Mobile phase: methanol/0.5% aqueous solution of acetic acid (92 : 8, v/v). flow rate: 1.0 mL min^−1^; detection: 310 nm.

Fig. 4A shows a schematic diagram of the online-storage inner-recycling CCC process. Initially, Fr. I and Fr. II were collected in test tubes in a one-step separation (Fig. 4B). Fr. III was then introduced into the storage loop by switching the six-port valve 2 (Fig. 4C). After all of Fr. III had been collected, six-port valve 2 was turned back and six-port valve 1 was switched to enable inner-recycling CCC separation of Fr. IV (Fig. 4D). After six cycles of separation with the solvent system *n*-heptane/ethyl acetate/methanol/acetic acid (5 : 4 : 1 : 1, v/v), 56.8 mg of compound 7 and 16.4 mg of compound 8 were obtained, each with purity >98%, as determined by HPLC (Fig. 5). Fig. 4E shows the inner-recycling CCC separation of Fr. III by switching six-port valves 1 and 2. After nine cycles of separation, 19.7 mg of compound 4 and a mixture of compounds 5 and 6 were obtained.

The mixture of compounds 5 and 6 was further purified by preparative HPLC, eluting with methanol/0.5% aqueous acetic acid (92 : 8, v/v) at a flow rate of 3.0 mL min^−1^ with monitoring at 310 nm. Compounds 5 (53.8 mg) and 6 (10.9 mg) were thereby obtained, each with purity >98%, as determined by HPLC (Fig. 5).

Fig. 5 shows the HPLC chromatograms of the crude extract and the isolated compounds. As shown in the amplification of Fig. 5A, peak 3 and peak 4 were eluted at nearly the same time in the HPLC column. This means that these two compounds were not well separated in pre-HPLC due to their similar polarities. As a liquid–liquid partition chromatography, HSCCC can separate alkylphenols in different dimensions. The inner-recycling CCC mode has many advantages, including low solvent consumption, high separation efficiency, and simple set-up. Its main drawback is peak broadening after many cycles of inner-recycling separation. If the front peak and the peak behind it are closely connected, this can be solved by releasing the eluents from the front and end of the peaks. We then turned to evaluating the inner-recycling CCC mode and the prediction of its efficacy by formula derivation.

### 3.3 Structure identification

#### 3.3.1 Compound 1 (peak 1 in Fig. 5B)

ESI-MS *m*/*z*: 317 [M − H]^−^. ^1^H NMR (400 MHz, CDCl_3_): *δ*_H_ 6.23 (2H, d, *J* = 2.3 Hz, H-4, 6), 6.17 (1H, d, *J* = 2.3 Hz, H-2), 5.35 (2H, m, CH=CH), 2.46 (2H, m, H-1′), 2.01 (4H, m, CH̲_2_–CH=CH–CH̲_2_), 1.55 (2H, m, H-2′), 1.29 (16H, m), 0.87 (3H, t, *J* = 6.7 Hz, CH_3_). ^13^C NMR (CDCl_3_, 100 MHz): *δ*_C_ 156.8 (C-1, 3), 146.0 (C-5), 130.0, 107.9 (C-4, 6), 100.2 (C-3), 35.9 (C-1′), 31.8, 31.1 (C-2′, 3′), 29.8, 29.7, 29.4, 29.3, 29.2, 29.0, 27.2 (CH_2_), 22.7 (C-14′), 14.1 (C-15′). Thus, the structure of 6 was determined as bilobol (15 : 1) by comparison of its MS, ^1^H and ^13^C NMR data with literature data.^29^

#### 3.3.2 Compound 4 (peak 4 in Fig. 5D)

ESI-MS *m*/*z*: 319 [M − H]^−^. ^1^H NMR (400 MHz, CDCl_3_): *δ*_H_ 7.34 (1H, dd, *J* = 8.2, 7.4 Hz, H-4), 6.85 (1H, d, *J* = 8.2 Hz, H-3), 6.75 (1H, d, *J* = 7.4 Hz, H-5), 2.96 (2H, t, *J* = 7.8 Hz, H-1′), 1.59 (2H, m, H-2′), 1.25 (20H, m), 0.88 (3H, t, *J* = 6.8 Hz, CH_3_). ^13^C NMR (CDCl_3_, 100 MHz): *δ*_C_ 174.7 (COOH), 163.6 (C-2), 147.5 (C-6), 134.9 (C-4), 122.5 (C-5), 115.7 (C-3), 110.8 (C-1), 36.5 (C-1′), 32.1, 31.9 (C-2′, 3′), 29.8, 29.7, 29.6, 29.5, 29.4 (CH_2_), 22.7 (C-12′), 14.1 (C-13′). Thus, the structure of 2 was determined as ginkgoneolic acid (GA 13:0) by comparison of its MS, ^1^H and ^13^C NMR data with literature data.^30^

#### 3.3.3 Compound 5 (peak 1 in Fig. 5E)

ESI-MS *m*/*z*: 347 [M − H]^−^. ^1^H NMR (400 MHz, CDCl_3_): *δ*_H_ 7.35 (1H, dd, *J* = 8.2, 7.4 Hz, H-4), 6.86 (1H, d, *J* = 8.2 Hz, H-3), 6.76 (1H, d, *J* = 7.4 Hz, H-5), 2.95 (2H, t, *J* = 7.6 Hz, H-1′), 1.60 (2H, m, H-2′), 1.26 (24H, m), 0.88 (3H, t, *J* = 6.8 Hz, CH_3_). ^13^C NMR (CDCl_3_, 100 MHz): *δ*_C_ 175.7 (COOH), 163.5 (C-2), 147.4 (C-6), 134.8 (C-4), 122.6 (C-5), 115.6 (C-3), 110.6 (C-1), 36.5 (C-1′), 32.0, 31.8 (C-2′, 3′), 29.8, 29.7, 29.7, 29.5, 29.4 (CH_2_), 22.6 (C-14′), 14.0 (C-15′). Thus, the structure of 5 was determined as ginkgolic acid (GA 15:1) by comparison of its MS, ^1^H and ^13^C NMR data with literature data.^30^

#### 3.3.4 Compound 6 (peak 1 in Fig. 5F)

ESI-MS *m*/*z*: 345 [M − H]^−^. ^1^H NMR (400 MHz, CDCl_3_): *δ*_H_ 7.34 (1H, dd, *J* = 8.2, 7.4 Hz, H-4), 6.86 (1H, d, *J* = 8.2 Hz, H-3), 6.76 (1H, d, *J* = 7.4 Hz, H-5), 5.35 (2H, m, CH=CH), 2.97 (2H, m, H-1′), 2.01 (4H, m, CH̲_2_–CH=CH–CH̲_2_), 1.59 (2H, m, H-2′), 1.29 (16H, m), 0.87 (3H, t, *J* = 6.8 Hz, CH_3_). ^13^C NMR (CDCl_3_, 100 MHz): *δ*_C_ 175.8 (COOH), 163.5 (C-2), 147.7 (C-6), 135.2 (C-4), 130.3, 129.9 (C=C), 122.7 (C-5), 115.8 (C-3), 110.8 (C-1), 36.4 (C-1′), 32.0, 31.8 (C-2′, 3′), 29.8, 29.7, 29.4, 29.3, 29.0, 27.2 (CH_2_), 22.7 (C-14′), 14.1 (C-15′). Thus, the structure of 1 was determined as ginkgolic acid C17:2 (GA 17:2) by comparison of its MS, ^1^H and ^13^C NMR data with literature data.^30^

#### 3.3.5 Compound 7 (peak 1 in Fig. 5G)

ESI-MS *m*/*z*: 373 [M − H]^−^. ^1^H NMR (400 MHz, CDCl_3_): *δ*_H_ 7.33 (1H, dd, *J* = 8.2, 7.4 Hz, H-4), 6.87 (1H, d, *J* = 8.2 Hz, H-3), 6.77 (1H, d, *J* = 7.4 Hz, H-5), 5.34 (2H, m, CH=CH), 2.98 (2H, m, H-1′), 2.01 (4H, m, CH̲_2_–CH=CH–CH̲_2_), 1.60 (2H, m, H-2′), 1.30 (20H, m), 0.89 (3H, t, *J* = 6.7 Hz, CH_3_). ^13^C NMR (CDCl_3_, 100 MHz): *δ*_C_ 176.2 (COOH), 163.5 (C-2), 147.8 (C-6), 135.3 (C-4), 129.9, 129.8 (C=C), 122.7 (C-5), 115.8 (C-3), 110.9 (C-1), 36.5 (C-1′), 32.0, 31.8 (C-2′, 3′), 29.80, 29.76, 29.69, 29.64, 29.59, 29.53, 29.35, 29.01, 27.24 (CH_2_), 22.7 (C-16′), 14.1 (C-17′). Thus, the structure of 4 was determined as hydroginkgolic acid (GA 15:0) by comparison of its MS, ^1^H and ^13^C NMR data with literature data.^30^

#### 3.3.6 Compound 8 (peak 1 in Fig. 5H)

ESI-MS *m*/*z*: 371 [M − H]^−^. ^1^H NMR (400 MHz, CDCl_3_): *δ*_H_ 7.31 (1H, dd, *J* = 8.2, 7.4 Hz, H-4), 6.85 (1H, d, *J* = 8.2 Hz, H-3), 6.75 (1H, d, *J* = 7.4 Hz, H-5), 5.35 (4H, m, CH=CH), 2.95 (2H, m, H-1′), 2.77 (2H, m, =CH–CH̲_2_–CH=), 2.03 (4H, m, CH̲_2_–CH=CH–CH_2_–CH=CH–CH̲_2_), 1.57 (2H, m, H-2′), 1.32 (14H, m), 0.88 (3H, t, *J* = 6.7 Hz, CH_3_). ^13^C NMR (CDCl_3_, 100 MHz): *δ*_C_ 176.8 (COOH), 163.3 (C-2), 147.4 (C-6), 135.0 (C-4), 130.2, 130.1, 128.0, 127.9 (2C=C), 122.6 (C-5), 115.7 (C-3), 110.1 (C-1), 36.4 (C-1′), 32.0, 31.5, 29.8, 29.7, 29.4, 29.4, 29.3, 27.2, 27.2 (CH_2_), 25.6 (=CH–CH_2_–CH=), 22.6 (C-16′), 14.1 (C-17′). Thus, the structure of 3 was determined as ginkgolic acid C17:1 (GA 17:1) by comparison of its MS, ^1^H and ^13^C NMR data with literature data.^30^

### 3.4 Evaluation and prediction of inner-recycling CCC mode by formula derivation

According to basic chromatographic theory, the partition efficiency of a separation column may be evaluated by computing the number of theoretical plates (*N*) for each peak and the resolution (*R*_s_) between the peaks according to the following equations:

2 *N* = (4*t*_R_/*W*)^2^

3 *R*_s_ = 2(*t*_R_2__ − *t*_R_1__)/(*W*_1_ + *W*_2_)

where *t*_R_ is the retention time and *W* is the baseline peak width.

In HSCCC separation, the separation factor (*α*) and stationary phase retention (*S*_f_) can be described by eqn (4) and (6), respectively:

4 *α* = *K*_2_/*K*_1_

5 *V*_C_ = *V*_M_ + *V*_S_

6 *S*_f_ = *V*_S_/*V*_C_

7 *V*_S_ = *S*_f_*V*_C_

8 *t*_R_ = *V*_M_ + *K*_i_*V*_S_

where *K*_1_ and *K*_2_ are the *K*_D_ values of the adjacent chromatographic peaks in HSCCC (*K*_2_ > *K*_1_), and *V*_M_, *V*_S_, and *V*_C_ are the volumes of the mobile phase and stationary phase and the total volume of the CCC column, respectively. *K*_i_ is the *K*_D_ value of any peak i. Eqn (6) can be transformed to eqn (7).

9 *A* = *α*^*n*^ = (*K*_2_/*K*_1_)^*n*^

10 *R*_S_ = 2(*t*_R_2__ − *t*_R_1__)/(*W*_1_ + *W*_2_)

11 *R*_S_ = 2(*K*_2_ − *K*_1_)*V*_S_/(*W*_1_ + *W*_2_)

where *A* is the separation factor after *n* cycles of CCC separation. Eqn (10) can be transformed into eqn (11) by combining it with eqn (6). Considering the expansion coefficient (*a*) of the peak broadening, the expression for resolution (*R*_s_) was finally transformed into eqn (12).

12 *R*_S_ = [2*S*_f_*V*_C_(*α*^*n*^ − 1)*K*_1_^*n*^]/[(*W*_1_ + *W*_2_)*an*]

For successful accomplishment of the inner-recycling CCC mode, the width of peak 1 (*W*_1_) plus peak 2 (*W*_2_) must be less than or equal to the total volume (*V*_C_). When the width of peak 1 (*W*_1_) plus peak 2 (*W*_2_) is equal to the total volume (*V*_C_), eqn (12) can be transformed into eqn (13) and eqn (3) into eqn (14).

13 *R*_S_ = 2*S*_f_(*α*^*n*^ − 1)*K*_1_^*n*^/(*an*)

14 *R*_S_ = 2*n*(*t*_R_2__ − *t*_R_1__)/[(*W*_1_ + *W*_2_)*a*^*n*−1^]

If *R*_S_ = 1, the adjacent chromatographic peaks can be 98% separated. They may be completely separated with a resolution of 1.5. As can be seen from eqn (14), *R*_S_ depends on the expansion coefficient (*a*) and the number of separation cycles (*n*).

As can be seen in Table 1, *A*_1_ = *α*_1_^*n*^ = (*K*_3_/*K*_2_)^*n*^ = (0.90/0.89)^*n*^ = 1.01^*n*^, *A*_2_ = *α*_2_^*n*^ = (*K*_1_/*K*_3_)^*n*^ = (0.96/0.90)^*n*^ = 1.07^*n*^, and *A*_3_ = *α*_3_^*n*^ = (*K*_4_/*K*_5_)^*n*^ = (1.53/1.41)^*n*^ = 1.09^*n*^. In view of the base numbers in *A*_1_, *A*_2_, and *A*_3_, it would be difficult to separate compounds 2 and 3.

## 4 Conclusion

A novel and effective online-storage inner-recycling CCC method for separating complex compounds with similar *K*_D_ values has been applied to the sarcotesta of *G. biloba*. Through a combination of online-storage and recycling modes, coupled with preparative HPLC, ginkgolic acids have been successfully separated with high purity. This novel method has been evaluated and predicted by formula derivation. It has the advantages of low solvent consumption and enhanced separation efficiency compared with the conventional one-step CCC mode, making it especially well-suited for the separation of complex natural products.

## Conflicts of interest

There are no conflicts to declare.

## Supplementary Material

## References

[cit1] Boonkaew T., Camper N. (2005). Biological activities of Ginkgo extracts. Phytomedicine.

[cit2] Mazza M., Capuano A., Bria P., Mazza S. (2006). *Ginkgo biloba* and donepezil: a comparison in the treatment of Alzheimer's dementia in a randomized placebo-controlled double-blind study. Eur. J. Neurol..

[cit3] Bastianetto S., Ramassamy C., Doré S., Christen Y., Poirier J., Quirion R. (2000). The *Ginkgo biloba* extract (EGb 761) protects hippocampal neurons against cell death induced by β-amyloid. Eur. J. Neurosci..

[cit4] Strømgaard K., Nakanishi K. (2004). Chemistry and biology of terpene trilactones from *Ginkgo biloba*. Angew. Chem., Int. Ed..

[cit5] Hecker H., Johannisson R., Koch E., Siegers C. (2002). *In vitro* evaluation of the cytotoxic potential of alkylphenols from *Ginkgo biloba* L.. Toxicology.

[cit6] Baek S. H., Ko J., Lee J. H., Kim C., Lee H., Nam D., Lee J., Lee S., Yang W. M., Um J., Sethi G., Ahn K. S. (2017). Ginkgolic acid inhibits invasion and migration and TGF-β-induced EMT of lung cancer cells through PI3K/Akt/mTOR inactivation. J. Cell. Physiol..

[cit7] Wu L., Jiang X., Shen Y., Lu Z., Tu G., Fu X., Chen S., Cao J. (2011). Efficacy of ginkgolic acids against Cryptosporidium andersoni in cell culture. Parasitol. Res..

[cit8] Wang G., Jiang D., Zhou Z., Zhao Y., Shen Y. (2009). *In vivo* assessment of anthelmintic efficacy of ginkgolic acids (C13:0, C15:1) on removal of Pseudodactylogyrus in European eel. Aquaculture.

[cit9] Lee J., Kim Y., Ryu S. Y., Cho M. H., Lee J. (2014). Ginkgolic acids and *Ginkgo biloba* extract inhibit *Escherichia coli* O157:H7 and Staphylococcus aureus biofilm formation. Int. J. Food Microbiol..

[cit10] Oh J., Hwang I. H., Hong C., Lyu S., Na M. (2013). Inhibition of fatty acid synthase by ginkgolic acids from the leaves of *Ginkgo biloba* and their cytotoxic activity. J. Enzyme Inhib. Med. Chem..

[cit11] Fu Y., Hong S., Li D., Liu S. (2013). Novel chemical synthesis of ginkgolic acid (13 : 0) and evaluation of its tyrosinase inhibitory activity. J. Agric. Food Chem..

[cit12] Fukuda I., Ito A., Hirai G., Nishimura S., Kawasaki H., Saitoh H., Kimura K., Sodeoka M., Yoshida M. (2009). Ginkgolic acid inhibits protein SUMOylation by blocking formation of the E1-SUMO intermediate. Chem. Biol..

[cit13] Sun Y., Tang C., Wu X., Pan Z., Wang L. (2012). Characterization of alkylphenol components in *Ginkgo biloba* Sarcotesta by thermochemolysis-gas chromatography/mass spectrometry in the presence of trimethylsulfonium hydroxide. Chromatographia.

[cit14] Beek T. A., Montoro P. (2009). Chemical analysis and quality control of *Ginkgo biloba* leaves, extracts, and phytopharmaceuticals. J. Chromatogr. A.

[cit15] Itokawa H., Totsuka N., Nakahara K., Takeya K., Lepoittevin J., Asakawa Y. (1987). Antitumor principles from *Ginkgo biloba* L. Chem. Pharm. Bull..

[cit16] Nagabhushana K. S., Ravindranath B. (1995). Efficient medium-scale chromatographic group separation of anacardic acids from solvent-extracted cashew nut (Anacardium occidentale) shell liquid. J. Agric. Food Chem..

[cit17] Li R., Shen Y., Zhang X., Ma M., Chen B., Beek T. A. (2014). Efficient purification of ginkgolic acids from *Ginkgo biloba* leaves by selective adsorption on Fe_3_O_4_ magnetic nanoparticles. J. Nat. Prod..

[cit18] Wang Y., Zhang L., Guo X., Wu S. (2018). Salting-in counter-current chromatography separation of tanshinones based on room temperature ionic liquids. J. Chromatogr. A.

[cit19] Liu Y., Kuang P., Guo S., Sun Q., Xue T., Li H. (2018). An overview of recent progress in solvent systems, additives and modifiers of counter current chromatography. New J. Chem..

[cit20] Gao Q. P., Ma R. Y., Chen L., Shi S. Y., Cai P., Zhang S. H., Xiang H. Y. (2017). Antioxidant profiling of vine tea (*Ampelopsis grossedentata*): Off-line coupling heart-cutting HSCCC with HPLC-DAD-QTOF-MS/MS. Food Chem..

[cit21] Zou D., Zhu X., Zhang F., Du Y., Ma J., Jiang R. (2018). An efficient strategy based on liquid-liquid extraction with three-phase solvent system and high speed counter-current chromatography for rapid enrichment and separation of epimers of minor bufadienolide from toad meat. J. Agric. Food Chem..

[cit22] Zou D., Du Y., Kuang J., Sun S., Ma J., Jiang R. (2018). pH-zone-refining counter-current chromatography with a hydrophilic organic/salt-containing two-phase solvent system for preparative separation of polar alkaloids from natural products. J. Chromatogr. A.

[cit23] Leitão S., Leitão G., Vicco D., Pereira J., Simão G., Oliveira D., Celano R., Campone L., Piccinelli A., Rastrelli L. (2017). Counter-current chromatography with off-line detection by ultra high performance liquid chromatography/high resolution mass spectrometry in the study of the phenolic profile of Lippia origanoides. J. Chromatogr. A.

[cit24] Dai C., Liu M., Zhang W., Lam C., Guo J., Li W., Wu J., Chen J., Chen Z., Zhang W., Yao M. (2017). A material-basis study of Aloe vera on the wnt/β-catenin signaling pathway using a knockin/knockout method with high-speed countercurrent chromatography. RSC Adv..

[cit25] Sun Z., He J., Lan J., Mu Q. (2017). High-speed counter-current chromatography with an online storage technique for the preparative isolation and purification of dihydroflavonoids from *Sophora alopecuroides L*.. Phytochem. Anal..

[cit26] Chen B., Liu Z., Zhang Y., Li W., Sun Y., Wang Y., Wang Y., Sun Y. (2018). Application of high-speed counter-current chromatography and HPLC to separate and purify of three polyacetylenes from *Platycodon grandiflorum*. J. Sep. Sci..

[cit27] Zhu H., Wang D., Wen L., Yu J., Geng Y., Zhao H., Zhao R., Xiao W. (2016). Preparative separation of quaternary ammonium alkaloids from Caulis Mahoniae by conventional and pH-zone-refining counter-current chromatography. RSC Adv..

[cit28] Yang Y., Yang J., Fang C., Gu D., Ma Y., Ito Y. (2018). A multilayer coil in type-I counter-current chromatography. J. Chromatogr. A.

[cit29] Suzuki Y., Esumi Y., Hyakutake H., Kono Y., Sakurai A. (1996). Isolation of 5-(8′Z-heptadecenyl)-resorcinol from etiolated rice seedlings as an antifungal agent. Phytochemistry.

[cit30] Beek T. A., Wintermans M. S. (2001). Preparative isolation and dual column high-performance liquid chromatography of ginkgolic acids from *Ginkgo biloba*. J. Chromatogr. A.

